# Effects of prolonged endotoxemia on liver, skeletal muscle and kidney mitochondrial function

**DOI:** 10.1186/cc5013

**Published:** 2006-08-08

**Authors:** Francesca Porta, Jukka Takala, Christian Weikert, Hendrik Bracht, Anna Kolarova, Bernhard H Lauterburg, Erika Borotto, Stephan M Jakob

**Affiliations:** 1 Department of Intensive Care Medicine, University Hospital Bern, Switzerland; 2 Department of Clinical Pharmacology, University Hospital Bern, Switzerland

## Abstract

**Introduction:**

Sepsis may impair mitochondrial utilization of oxygen. Since hepatic dysfunction is a hallmark of sepsis, we hypothesized that the liver is more susceptible to mitochondrial dysfunction than the peripheral tissues, such as the skeletal muscle. We studied the effect of prolonged endotoxin infusion on liver, muscle and kidney mitochondrial respiration and on hepatosplanchnic oxygen transport and microcirculation in pigs.

**Methods:**

Twenty anesthetized pigs were randomized to receive either endotoxin or saline infusion for 24 hours. Muscle, liver and kidney mitochondrial respiration was assessed. The cardiac output (thermodilution) and the carotid, superior mesenteric and kidney arterial, portal venous (ultrasound Doppler) and microcirculatory blood flow (laser Doppler) were measured, and systemic and regional oxygen transport and lactate exchange were calculated.

**Results:**

Endotoxin infusion induced hyperdynamic shock and impaired the glutamate-dependent and succinate-dependent mitochondrial respiratory control ratio in the liver (glutamate, median (range) endotoxemia 2.8 (2.3–3.8) vs controls 5.3 (3.8–7.0); *P *< 0.001; succinate, endotoxemia 2.9 (1.9–4.3) vs controls 3.9 (2.6–6.3), *P *= 0.003). While the ADP added/oxygen consumed ratio was reduced with both substrates, the maximal ATP production was impaired only in the succinate-dependent respiration. Hepatic oxygen consumption and extraction, and the liver surface laser Doppler blood flow remained unchanged. Glutamate-dependent respiration in the muscle and kidney was unaffected.

**Conclusion:**

Endotoxemia reduces the efficiency of hepatic mitochondrial respiration but neither skeletal muscle nor kidney mitochondrial respiration, independent of regional and microcirculatory blood flow changes.

## Introduction

Organ dysfunction is a hallmark of severe sepsis despite normal or high systemic oxygen delivery [1]. The hepatosplanchnic organs are susceptible to insufficient perfusion in severe sepsis and septic shock, and hepatic function is impaired even in hemodynamically stable sepsis [2]. Although microvascular blood flow abnormalities have been described in experimental and human sepsis [3,4], it is unlikely that these alone would explain the pathogenesis of hepatic dysfunction. Rather, changes in cellular metabolism – specifically utilization of oxygen – are likely to contribute. The concept of sepsis-induced abnormalities in oxygen utilization is supported by findings of elevated tissue oxygen tension [5,6] and decreased oxygen consumption [7], together with functional and biochemical derangements but minimal cell death [8] in sepsis and septic shock.

Several authors [9-13] have reported alterations in oxygen utilization at the mitochondrial level during experimental sepsis, and differences in organ sensitivity have been described [14]. In rats, nitric oxide overproduction, complex I inhibition and ATP depletion were observed in liver and skeletal muscle mitochondria in severe sepsis [12]. In rabbits, the mitochondrial state 3 respiration was reduced after endotoxin administration in cardiac and skeletal muscle [13]. In rats, heart mitochondrial respiration but not kidney mitochondrial respiration was impaired after 6 hours of endotoxin infusion [14]. Other workers have also reported endotoxin-induced inhibition of the mitochondrial respiratory chain enzyme complexes [9,10]. Furthermore, endotoxin may induce mitochondrial structural alterations, leading to oxygen waste through the inner mitochondrial membrane [9,11]. In humans, skeletal muscle mitochondrial dysfunction has been related to severity of sepsis and poor outcome [15].

Although mitochondrial dysfunction has been found in the presence of normal or high oxygen delivery, it is conceivable that previous and/or concomitant insufficient tissue perfusion may contribute to changes in mitochondrial respiration. Since the microcirculatory blood flow is heterogeneous in septic states [4], tissue units with adequate and insufficient perfusion may coexist. In addition, the hepatosplanchnic region is hypermetabolic in sepsis, making it susceptible to insufficient perfusion [2]. It has recently been suggested that the analysis of muscle mitochondrial function may be used as an indicator of mitochondrial alterations in other vital organs [12].

We hypothesized that endotoxemia has different effects on mitochondrial function in the liver compared with function in the peripheral tissues, such as the skeletal muscle. Moreover, if endotoxin-induced abnormalities in the microcirculatory blood flow and consecutive impairment of regional oxygen availability contribute to mitochondrial dysfunction, this should be more evident in organs with a relatively high oxygen extraction, such as the liver, than in organs with a low oxygen extraction, such as the kidney.

We therefore compared the effects of prolonged endotoxemia on mitochondrial function in the liver, kidney and skeletal muscle in pigs. We furthermore evaluated the concomitant changes in hepatosplanchnic oxygen transport, the hepatic redox state and microcirculation.

## Materials and methods

The study was performed in accordance with the National Institutes of Health guidelines for the care and use of experimental animals and with the approval of the Animal Care Committee of the Canton of Berne, Switzerland. Preliminary results on hemodynamics, lactate exchange and mitochondrial respiratory function from two of the pigs included in this study have been published previously [16]. The experimental setting, the surgical preparation and instrumentation and the fluid management have been described in detail previously [16].

Briefly, 20 pigs (37–42 kg) were fasted overnight, premedicated with ketamine (20 mg/kg) and xylazine (2 mg/kg intramuscularly), followed by intravenous administration of midazolam (0.5 mg/kg) and atropine (0.02 mg) for endotracheal intubation. The animals were ventilated with a volume-controlled ventilator (Servo ventilator 900 C; Siemens, Elema, Sweden) with 5 cmH_2_O end-expiratory pressure. The FiO_2 _was adjusted to keep PaO_2 _levels between 13.3 kPa (100 mmHg) and 20 kPa (150 mmHg), and the minute ventilation was adjusted to maintain PaCO_2 _levels between 4.5 and 5.5 kPa (34–41 mmHg). Before the start of the abdominal operation, muscle samples (6–8 g) were excised from the quadriceps muscle in 12 animals and the mitochondria were rapidly isolated in order to test the respiratory activity. During surgery, the animals received normal saline at a rate of 8 ml/kg/hour. Anesthesia was maintained with thiopental (7 mg/kg/hour) and fentanyl (3 μg/kg/hour).

After performance of a midline abdominal incision, ultrasound Doppler flow probes (Transonic^® ^System Inc., Ithaca, NY, USA) that had been calibrated *in vitro *were installed for measurement of blood flow in the superior mesenteric, hepatic, and renal arteries and in the portal vein. Microcirculatory blood flow was measured using pre-calibrated laser Doppler flow probes (Oxford Optronix, Oxford, UK) sutured onto the surface of the liver and the kidney.

Carotid and pulmonary arterial, central venous, hepatic venous and portal venous blood pressures and the pulmonary artery occlusion pressure were recorded with quartz pressure transducers. The cardiac output was measured by the thermodilution technique (S/5 Compact Critical Care monitor; Datex-Ohmeda, Helsinki, Finland). The central temperature was recorded from the thermistor in the pulmonary artery catheter (CO/SvO_2 _catheter; Edwards Lifesciences, Munich, Germany). The heart rate and the electrocardiogram were continuously monitored. Once the experiment was started, manipulation was avoided to minimize the possibility of flow probe displacement. At the end of the experiment, the correct position of each probe was controlled visually.

### Experimental protocol

After preparation, 180 minutes were allowed for hemodynamic stabilization. The animals were then randomized into two groups (*n *= 10 per group): a control group with saline infusion, and an experimental group with endotoxin infusion for 24 hours or until death of the animal.

Endotoxin was infused into the right atrium (*Escherichia coli *lipopolysaccharide B0111:B4, 20 mg/l in 5% dextrose; Difco Laboratories, Detroit, MI, USA). The initial infusion rate was 0.4 μg/kg/hour until the mean pulmonary arterial pressure reached 30 mmHg. The infusion was then stopped and subsequently adjusted to maintain moderate pulmonary artery hypertension (mean pulmonary artery pressure, 25–30 mmHg), unless the mean systemic artery pressure decreased below 50 mmHg with no response to additional fluids. If hypotension persisted, the endotoxin infusion was temporarily stopped. Gelatin (Physiogel 4%; Braun, Emmenbrücke, Switzerland) was administered as required to maintain the pulmonary artery occlusion pressure between 5 and 8 mmHg. While the primary target variable in fluid management was the pulmonary artery occlusion pressure, additional volume boluses of 50 ml were given as long as the stroke volume increased if hypovolemia was suspected despite the target filling pressure being reached (hypotension, tachycardia, oliguria, increase in arterial lactate concentration). Glucose (50%) was administered in order to maintain a blood glucose concentration between 3.5 and 6 mmol/l.

After evaluation of the dose response to endotoxin in a pilot phase, we found that 0.4 μg/kg/hour was the optimal endotoxin dose to induce long-term septic shock with a mean arterial pressure above 50 mmHg. At the end of the experiment, tissue samples were taken for isolation of mitochondria from the liver, muscle and kidney. For technical reasons, one sample of the liver and two samples from the skeletal muscle from endotoxemic animals were not available for analysis. In addition, samples for the study of kidney mitochondria were taken in the last 15 experiments only (control, *n *= 8; endotoxemia, *n *= 7). Tissue samples for mitochondrial function assessment were always obtained before death occurred. The animals were sacrificed with an overdose of intravenous potassium chloride.

### Liver mitochondrial isolation

Isolation of liver mitochondria was performed at 4°C using a standard procedure based on differential centrifugation [17]. The samples of liver (6–8 g) excised at the end of the experiment were rapidly immersed in ice-cold isolation buffer (mannitol 220 mmol/l, sucrose 70 mmol/l, morpholinopropane sulfonic acid 5 mmol/l, pH 7.4), transported to the laboratory and weighed. Tissue was minced with scissors and homogenized with an additional 10 volumes (wt/vol) of homogenization media (isolation buffer plus ethyleneglycol tetraacetate 2 mmol/l) in a Potter Elvehjem homogenizer with a loose-fitting Teflon pestle (four strokes). The homogenate was then centrifuged for 10 minutes at 700 × *g*. The supernatant was collected and centrifuged again for 10 minutes at 7,000 × *g*. The supernatant was discarded at this time; the pellet was then resuspended in isolation buffer and centrifuged twice for 10 minutes at 7,000 × *g*, for further purification of the mitochondria. The pellets were then suspended in buffer at a final concentration of 50–100 mg mitochondrial protein per milliliter.

### Muscle mitochondrial isolation

Skeletal muscle mitochondria were isolated as described by Hoppel and colleagues [18]. Quadriceps muscle specimens were rapidly immersed in ice-cold isolation buffer (KCl 100 mmol/l, MgSO_4 _10 mmol/l, morpholinopropane sulfonic acid 50 mmol/l, ethylenedinitrolotetraacetic acid 1.0 mmol/l, ATP 1.1 mmol/l, pH 7.4), were transported to the laboratory and were weighed. After several rinses with isolation buffer, the skeletal muscle was minced using scissors and was suspended in 10 volumes (wt/vol) of the same medium and treated with a protease (Protease; Sigma-Aldrich, St Louis, MO, USA) 5 mg/g mince for 10 minutes at 4°C with constant stirring. The suspension was diluted with an equal volume of isolation medium supplemented to 0.2% (wt/vol) with defatted BSA and homogenized in a Potter Elvehjem homogenizer with a loose-fitting Teflon pestle (10 strokes). The supernatant was separated by centrifugation (10 min at 10,000 × *g*), and the pellet was resuspended in BSA-supplemented isolation medium (10 ml/g tissue). The suspension was centrifuged for 10 minutes at 2,500 × *g*, the supernatant was filtered through two layers of gauze, and the mitochondria were sedimented at 7,700 × *g *for 10 minutes. The mitochondria were subjected to two additional washes using 5 ml BSA-supplemented isolation medium/g tissue and 2.5 ml of KCl 100 mmol/l, morpholinopropane sulfonic acid 50 mmol/l, ethyleneglycol tetraacetate 0.5 mmol/l (pH 7.4/g muscle), and were finally resuspended in approximately1.0 ml of KCl 100 mmol/l, morpholinopropane sulfonic acid 50 mmol/l, ethylenglycol tetraacetate 0.5 (pH 7.4).

### Determination of mitochondrial respiration

The protein concentration was determined spectrophotometrically with the Biuret method using BSA as standard. For the analysis of the mitochondrial respiration, mitochondria were incubated in a 3 ml incubation chamber (Yellow Springs Instruments, Yellow Springs, OH, USA) at 30°C, in a medium consisting of KCL 25 mmol/l, morpholinopropane sulfonic acid 12.5 mmol/l, ethylene glycol-bis *N*,*N*,*N'*,*N' *-tetraacetic acid 1 mmol/l and potassium phosphate buffer 5 mmol/l (pH 7.4). Oxygen consumption was determined using a Clark-type electrode (Yellow Springs Instruments) adding one of the following respiratory substrates: glutamate 20 mmol/l to examine the complex I-dependent, complex II-dependent and complex IV-dependent respiration; succinate 20 mmol/l for complex II-dependent and complex IV-dependent respiration; and ascorbate 0.12 mmol/l/*N*,*N*,*N'*,*N' *-tertamethyl-*p*-phenyldiamine (TMPD) 0.24 mmol/l for complex IV-dependent respiration.

The mitochondrial respiratory function is conventionally separated into different states [19]. State 3 is defined as the ADP-dependent oxygen consumption and reflects the mitochondrial respiration coupled to ATP production. State 4, the resting respiration, is a measure of the oxygen consumed uncoupled from ATP synthesis, but required to maintain the integrity of the membrane potential. State 3 respiration rates were determined in the presence of ADP 200 μmol/l. The rates measured after the consumption of ADP were taken as the state 4 respiration rates. Oxygen consumption rates are expressed as nanoatom O_2 _per minute per milligram of protein. The respiratory control ratio (RCR) (state 3/state 4) for glutamate-dependent, succinate-dependent and ascorbate/TMPD-dependent respiration, and the ADP added/oxygen consumed (ADP:O) ratio (nanomol/nanoatom) for glutamate-dependent and succinate-dependent respirations were calculated according to Estabrook [20]. Since the transition from state 3 to state 4 occurs very slowly in ascorbate/TMPD-dependent respiration, the ADP:O ratio was not calculated. Finally, the maximal ATP production (ADP:O ratio * state 3 respiration, nanomol * nanoatom/minute/mg protein) for glutamate-dependent and succinate-dependent respiration was derived [21].

### Blood sampling

Blood samples for the measurement of hemoglobin, lactate and blood gases were taken at baseline, after 1.5, 3, 6 and 12 hours of endotoxin or saline infusion, and at the end of the experiment. The blood samples were taken from the pulmonary and carotid arteries, and from the portal, hepatic, mesenteric and kidney veins (ABL 520 and OSM 3 [pig module]; Radiometer, Copenhagen, Denmark). Pyruvate was measured by enzymatic color reaction (Sigma Diagnostics, St Louis, MO, USA) and spectrophotometrically (VERSAmax™; Molecular Devices Corporation, Sunnyvale, CA, USA) by changes in absorbance at 340 nm.

The following equations were used:

Oxygen content (ml/l) = hemoglobin (g/l) × oxygen saturation × 1.39 + 0.03 × pO_2 _(mmHg)

Oxygen delivery (DO_2_) (ml/minute/kg) = systemic or regional blood flow (l/minute/kg) × arterial oxygen content (ml/l)

Oxygen consumption (VO_2_) (ml/minute/kg) = systemic or regional blood flow (l/minute/kg) × (arterial oxygen content – venous oxygen content [ml/l])

Oxygen extraction ratio = VO_2 _/DO_2_

Lactate exchange (μmol/minute/kg) = regional lactate influx – regional lactate efflux

Lactate/pyruvate ratio = lactate (μmol/l)/pyruvate (μmol/l)

RCR = state 3 (nanoatom O_2_/minute/mg proteins)/state 4 (nanoatom O_2_/minute/mg proteins)

## Statistical analysis

The SPSS 11.0 software package (SPSS Inc., Chicago, IL, USA) was used for statistical analysis. All animals but one were included in the statistical analysis.

Since this model of endotoxemia has an expected mortality of 40–60% at 24 hours, hemodynamic, oxygen transport and lactate exchange data from the first 12 hours of the experimental protocol – when all animals were still alive – were analyzed first. The 12-hour values were then compared with the measurements at the end of the experiment (24 hours or death) when the biopsies were taken for the isolation of mitochondria. Changes within groups until 12 hours were assessed by the Friedman test. Changes between 12 hours and the end of the experiment were assessed by the Wilcoxon test. Differences between groups were assessed after 12 hours and at the end of the experiment using the Mann-Whitney *U *test. In five animals the effect of the prolonged anesthesia and surgery on the mitochondrial respiration was tested by comparing samples taken from the muscle of animals before the surgery and at the end of the experiment, using the Wilcoxon test.

Results are presented as the median (range). Statistical significance was considered at *P *< 0.05.

## Results

One animal in the control group developed refractory cardiac arrhythmia; the experiment therefore had to be terminated early, and data from this animal could not be included in the analysis.

At baseline, before administration of endotoxin or placebo (180 minutes after completing the surgery), there were no significant differences between the two groups in any of the measured variables.

**Figure 1:**
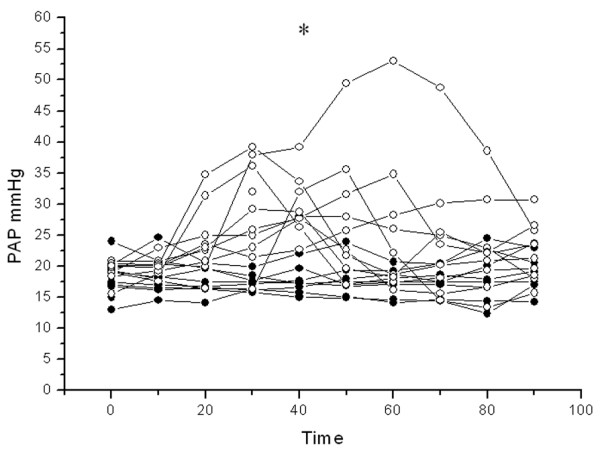
Pulmonary artery pressure changes during the first 90 minutes of infusion. Pulmonary artery pressure (PAP) changes during the first 90 minutes of placebo infusion (filled circles) or during endotoxin infusion (open circles). *Friedman test, *P *< 0.001 group effect. Only the maximum PAP value (recorded manually) was included for one animal because part of the continually recorded data were lost due to a technical failure.

**Figure 2:**
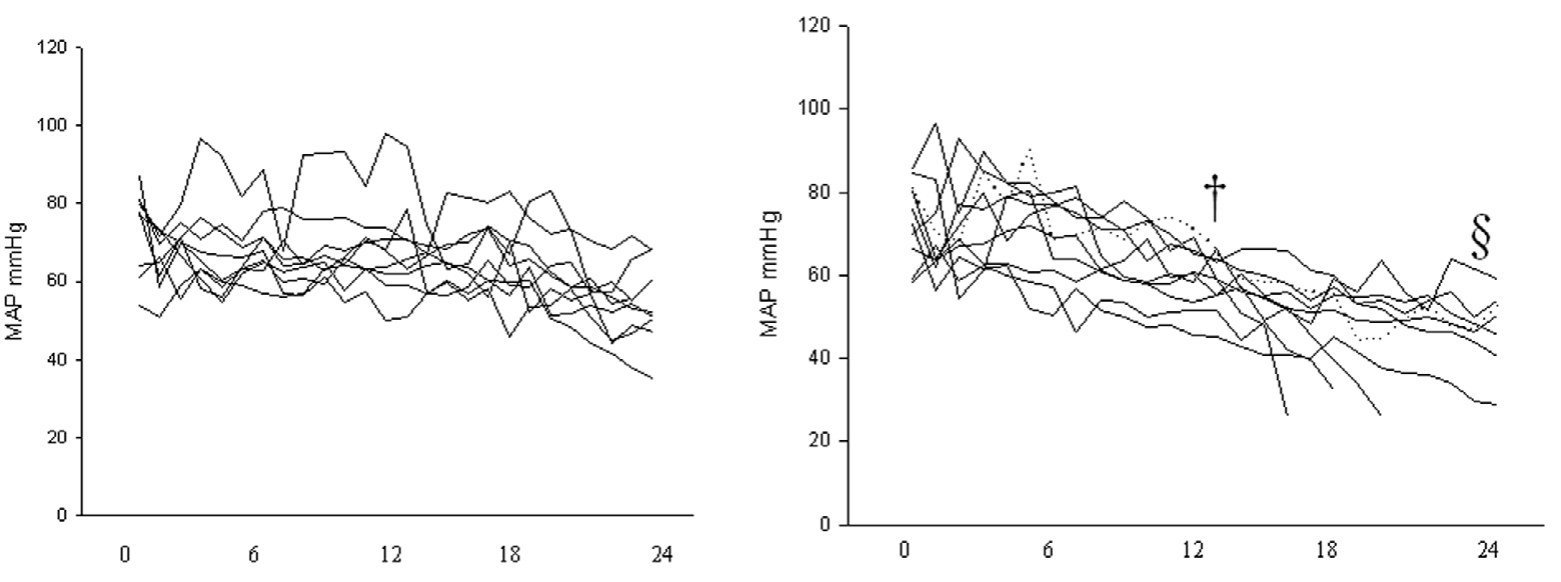
Mean arterial pressure changes over the course of the 24-hour experiment. Mean arterial pressure (MAP) changes in **(a) **the control group and **(b) **the endotoxin group. Dotted line, pig in which the biopsy samples were taken erroneously after 18 hours of the experiment. ^†^*P *= 0.001 12 hours vs baseline; ^§^*P *= 0.02 vs 12 hours.

**Figure 3:**
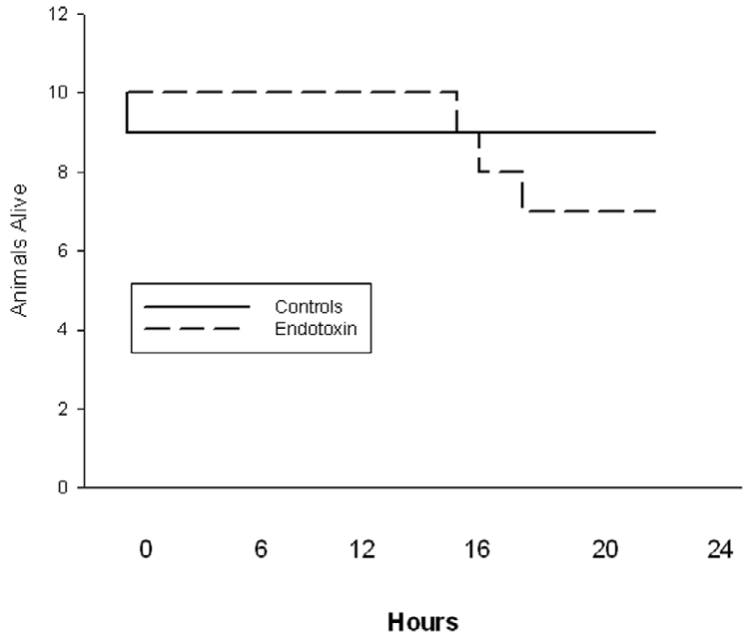
Mortality during the experiment.

Infusion of endotoxin induced early pulmonary artery hypertension (Figure 1), followed by systemic hypotension and increased cardiac output (Table 1). Three of the 10 endotoxemic animals died between 12 and 24 hours in severe shock with hypotension unresponsive to fluid administration (Figure 2), resulting in 30% mortality at 24 hours (Figure 3). Tissue samples were erroneously taken at 18 hours instead of 24 hours of endotoxin infusion in one endotoxin-infused animal. Data from this animal are labeled separately in Figure 2. The liver and kidney microcirculation could not be measured in one endotoxin-infused animal for technical reasons.

Endotoxin infusion was temporarily stopped in all the animals of the experimental group, owing to a severe pulmonary artery pressure increase and to systemic hypotension. The time of endotoxin discontinuation was 54 (22–410) minutes.

All the animals were alive at the time of the organ sampling. Hemodynamic and metabolic variables at this time of organ sampling are presented in Tables 1, 2, 3, 4 as 'End of experiment'.

### Liver

**Figure 4:**
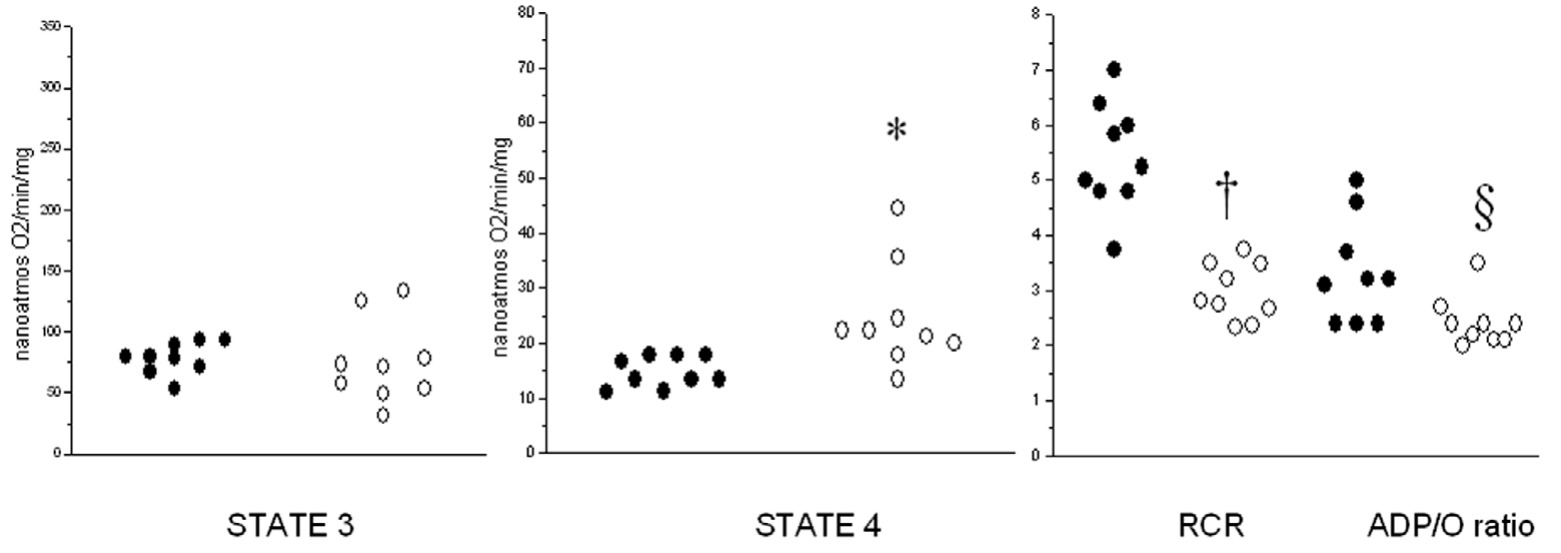
Glutamate-dependent respiration rates, respiratory control ratio and ADP added/oxygen consumed ratios in the liver. Glutamate-dependent state 3 and state 4 respiration rates (nanoatom/min/mg protein), the respiratory control ratio (RCR) and the ADP added/oxygen consumed (ADP:O) ratio (nanomol/nanoatom) in liver biopsies after placebo infusion (filled circles) and after endotoxin infusion (open circles). *P = 0.002 vs control group; ^†^*P *= 0.000 vs control group, ^§^*P *= 0.014 vs control group.

Endotoxin infusion increased the resting (state 4) glutamate-dependent respiration (*P *= 0.002), while mitochondrial glutamate-dependent state 3 respiration was not altered. The liver glutamate-dependent RCR consequently decreased (2.8 (2.3–3.8) in endotoxemia vs 5.3 (3.8–7.0) in controls, *P *< 0.001) (Figure 4). The stoichiometry of complex I, expressed by the ADP:O ratio, was impaired in the presence of endotoxin (Figure 4), while the decrease in maximal ATP production was not statistically significant (152 (74–303) nanomol * nanoatom/minute/mg protein in endotoxemia vs 248 (130–428) nanomol * nanoatom/minute/mg protein in controls, *P *= 0.07).

**Figure 5:**
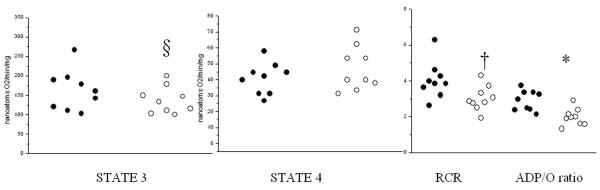
Succinate-dependent respiration rates, respiratory control ratio and ADP added/oxygen consumed ratios in the liver. Succinate-dependent state 3 and state 4 respiration rates (nanoatom/min/mg protein), the respiratory control ratio (RCR) and the ADP added/oxygen consumed (ADP:O) ratio (nanomol/nanoatom) in liver biopsies after placebo infusion (filled circles) and after endotoxin infusion (open circles). *P = 0.001 vs control group; †*P *= 0.003 vs control group; §*P *= 0.001 vs control group.

Endotoxin induced a decrease in succinate-dependent state 3 respiration (*P *= 0.001), while the state 4 respiration did not change. The respiratory efficiency (RCR) consequently decreased, together with both a reduced ADP:O ratio (Figure 5) and a maximal ATP production (262 (152–423) nanomol * nanoatom/minute/mg protein in the endotoxin-infused group vs 403 (266–667) nanomol * nanoatom/minute/mg protein in controls; *P *= 0.008). No changes over time or differences between groups were found in ascorbate/TMPD-dependent respiration rates, or in the RCR (1.6 (1.2–2.1) in endotoxin-infused animals vs 1.8 (1.1–3.3) in controls, not significant). The ascorbate/TMPD-dependent state 4 respiration in liver mitochondria was not clearly detectable in one endotoxemic animal, and consequently the RCR could not be calculated.

Endotoxin infusion had no significant effect on hepatic oxygen consumption and oxygen extraction, and the hepatic venous lactate/pyruvate (L/P) ratio remained unchanged for the first 12 hours (Table 4) and did not change significantly from 12 hours to the end of the experiment. Markedly elevated hepatic venous L/P ratios were observed in individual animals in the endotoxin group by the end of the experiment. The hepatic lactate uptake also reverted to hepatic lactate release in two of the three animals receiving endotoxin that died before the end of the experiment. The liver blood flow increased during the first 12 hours (Table 3). The liver surface laser Doppler blood flow was highly variable and did not change significantly (Table 3).

### Skeletal muscle

Endotoxin infusion had no effect on skeletal muscle glutamate-dependent, succinate-dependent or ascorbate/TMPD-dependent mitochondrial respiration, and consequently on the RCR (Table 2). The ADP:O ratios were stable in control animals and in endotoxin-infused animals (glutamate, 2.0 (1.7–3.0) nanomol/nanoatom in the endotoxin-infused group vs 2.5 (1.6–2.9) nanomol/nanoatom in the control group, not significant; succinate, 2.0 (1.6–2.3) nanomol/nanoatom in the endotoxin-infused group vs 2.3 (1.2–3.3) nanomol/nanoatom in the control group, not significant). The maximal ATP production after glutamate and succinate addition was not affected by endotoxin (Table 2).

The prolonged anesthesia and surgery had no effect on the skeletal muscle glutamate-associated, succinate-associated or ascorbate/TMPD-associated mitochondrial respiration. The preoperative state 3 respiration rate was 218 (100–280) nanoatom/minute/mg mitochondrial protein for glutamate-dependent respiration, was 171 (134–418) nanoatom/minute/mg for succinate-dependent respiration, and was 356 (209–503) nanoatom/minute/mg for ascorbate/TMPD-dependent respiration. The state 4 respiration rates were 25 (11–60) nanoatom/minute/mg, 62 (29–103) nanoatom/minute/mg and 178 (126–271) nanoatom/minute/mg, respectively. The RCRs were 8.4 (5.4–12.0) for glutamate, 4.2 (2.4–5.8) for succinate and 1.8 (1.1–2.8) for ascorbate/TMPD.

There were no significant differences in systemic oxygen transport-related variables between the groups at baseline (Table 3). Endotoxin had no effect on systemic oxygen consumption or on oxygen extraction (Table 3). The arterial lactate concentration and the L/P ratio remained unchanged for the first 12 hours (Table 4).

### Kidney

As already described in Materials and methods, the kidney mitochondrial respiration was analyzed in the last 15 animals (control, *n *= 8; endotoxin, *n *= 7) (Table 2). Two of the seven endotoxin-infused animals died between 12 and 24 hours.

Endotoxin infusion had no effect on renal glutamate-dependent, succinate-dependent or ascorbate/TMPD-dependent mitochondrial respiration, and consequently on the RCR (Table 2).

The ADP:O ratios were stable in control animals and in endotoxin-infused animals (glutamate, 2.6 (0.9–3.3) nanomol/nanoatom in the endotoxin-infused group vs 2.5 (1.4–3.7) nanomol/nanoatom in the control group, not significant; succinate, 2.3 (1.8–3.0) nanomol/nanoatom in the endotoxin-infused group vs 2.1 (1.5–5.1) nanomol/nanoatom in the control group, not significant). The maximal ATP production after glutamate and succinate addition was not affected by endotoxin (Table 2).

Kidney blood flow remained stable during the first 12 hours and decreased at the end of the experiment in endotoxin-infused animals (*P *= 0.03, Table 3). The renal oxygen extraction increased in endotoxemic animals from 24% (17–37%) at baseline to 29% (21–42%) after 12 hours (*P *= 0.001), resulting in an unchanged renal oxygen consumption. The renal oxygen extraction increased further until the end of the experiment in the endotoxin-infused animals (*P *= 0.012), without changes in the renal oxygen consumption (Table 3). The renal surface laser Doppler blood flow was highly variable in both groups, and decreased in the endotoxin-infused group at the end of the experiment (*P *= 0.036) (Table 3).

## Discussion

The main finding of this study was that prolonged endotoxemia impaired the efficiency of hepatic mitochondrial complex I and complex II respiration, whereas mitochondrial respiration in the skeletal muscle remained unchanged. The altered mitochondrial function occurred despite well-maintained total and microcirculatory hepatic blood flow. In spite of the reduced hepatic mitochondrial RCR, the hepatic oxygen consumption and extraction remained unchanged. The reduced glutamate-dependent RCR in the liver mitochondria was mainly due to an increase in the mitochondrial resting respiration rate, suggesting partial uncoupling of oxygen consumption from ATP production. These results are supported by the well-maintained hepatic oxygen consumption and by the reduction in the ADP:O ratios. The alterations in the succinate-dependent respiration were due to reduced function of the complex II, as suggested by reduced state 3 respiration. The partial uncoupling in the glutamate-dependent and succinate-dependent respirations, confirmed by the reduced ADP:O ratio and maximal ATP production in the latter, suggest alterations in the mitochondrial membrane integrity.

It has recently been suggested that muscle mitochondria could be used in clinical sepsis as markers of mitochondrial dysfunction in other, more vital, organs [12]. In a long-term model of fecal peritonitis, muscle and liver mitochondrial functions were impaired in rats with severe septic shock after 24 hours. The different animal model (rats versus pigs) and the type of sepsis (peritonitis versus endotoxemia) may partly explain the different results. In the rat model, however, the evaluation of sepsis severity was mostly based on clinical evaluation of the animals, and oxygen availability in the liver at the time of tissue sampling was not described. In this context, the potential role of organ hypoperfusion in mitochondrial function is uncertain. No specific data in human sepsis are available to evaluate the sensitivity and time course of mitochondrial function in different organs. Our results demonstrate that relevant differences between tissues exist in porcine endotoxemia. Furthermore, the liver appears to be more sensitive to endotoxin than the kidney and muscle. This could at least in part explain the findings of early impairment of hepatic function in clinical sepsis.

While species-specific differences are likely to exist, we used a long-term large-animal model, which, as compared with other experimental models (such as, in rodents), should more closely resemble clinical sepsis. In addition, organ-specific differences in response to endotoxin have also been found in other studies. Myocardial mitochondria in rabbits were more affected by endotoxin infusion than skeletal muscle mitochondria [13], and cardiac muscle respiration but not renal complex I-dependent state 3 respiration was decreased in neonatal rats during six hours of endotoxemia [22].

The sensitivity of the liver to endotoxemia is emphasized by the presence of hepatic mitochondrial dysfunction even in those pigs surviving to the end of the experiment without profound hypotension. In contrast, the normal skeletal muscle mitochondrial respiration even in pigs dying of severe shock suggests that, at least in pigs, the skeletal muscle mitochondria are resistant to the effects of endotoxin. Hepatic mitochondrial complex I function was impaired after endotoxin exposure despite preserved hepatic blood flow, oxygen consumption and extraction, and redox state, as indicated by the L/P ratio. Impaired function of isolated mitochondria in the presence of unlimited oxygen availability has been demonstrated earlier by our group [16] and by other workers [10], and can be explained with partial uncoupling of mitochondrial oxidative phosphorylation. Although many studies have demonstrated inhibition of mitochondrial complex function, isolated inefficiency in oxygen during septic states has also been reported by other studies [23]. Our results demonstrate *in vivo *that normal oxygen consumption and extraction can coexist with altered mitochondrial respiration.

The kidney mitochondrial function was not impaired by endotoxin infusion, despite a decrease in the kidney blood flow and impaired renal microcirculation. This finding, together with the liver mitochondrial alteration in the absence of flow or microcirculatory dysfunctions, suggests that endotoxin has a direct effect on liver cells which is not dependent on oxygen availability.

Although we did not measure ATP generation directly, it is possible that, despite normal oxygen extraction and consumption, energy production was insufficient for the hepatic metabolic needs during endotoxemia in the liver. The alteration in stoichiometric efficiency of complex I and complex II of the mitochondrial respiratory chain represents, by definition, a reduction in phosphate incorporation into ATP per amount of oxygen consumed, leading to a waste of redox energy and reduction of the proton gradient across the membrane. In this condition, the mitochondrial energy production is likely to be more dependent on oxygen supply, making the cells vulnerable to acute hemodynamic instability.

Endotoxin produced the characteristic hemodynamic response [24-27], with a rapid increase in pulmonary artery pressure [28] followed by increasing systemic blood flow and progressive hypotension, accompanied by flow redistribution between organs. In particular, the regional renal blood flow slightly decreased and the hepatic blood flow increased. Volume management in experimental sepsis may markedly modify the metabolic and hemodynamic responses. Our strategy, based on target filling pressures and the assessment of the response of the stroke volume to additional fluid boluses, resulted in relatively low total volumes of fluid administration as compared with some other models [29,30]. Had this been too restrictive, any impairment in oxygen utilization should have been more evident.

At the end of the experiment, the arterial blood pressure decreased moderately also in control animals despite a maintained cardiac output and stroke volume. We interpret this hypotension as an effect of prolonged anesthesia on vascular tone. The mitochondrial function in placebo-infused animals remained intact, confirming the role of endotoxin in the impairment of cellular function.

Our study has several limitations. The mitochondria population is not uniform within the organs. Senescent or damaged mitochondria are eventually removed and replaced within the lifetime of the cell, and the method we used for mitochondrial isolation is specifically designed to exclude mitochondria that are damaged or senescent. Since we did not directly investigate the mitochondrial structural integrity and the purity of our preparations, we cannot say to what extent our method underestimates the overall mitochondrial dysfunction after endotoxin exposure. The RCRs we obtained after endotoxin exposure, however, were comparable with those obtained using the same method by other groups [31].

During the measurement of the mitochondrial respiration, in order to avoid misinterpretation of the results, we excluded the use of inhibitors for the assessment of the complex II and complex IV respiration. We therefore cannot exclude the chance that we missed important findings about the function of these two complexes. Nevertheless, this did not influence the alteration we found in the complex-I-dependent respiration.

A methodological limitation of this study is the use of the laser Doppler technique to assess microcirculatory perfusion, since this method does not detect microcirculatory blood flow heterogeneity. We cannot exclude the presence of such alterations. Moreover, the flow probes were sutured onto the surface of the kidney and the liver, while the deeper tissue layers were not investigated. Nevertheless, the method is validated to give accurate estimates of the changes in flow velocity [32] and has already been used in septic models [33]. In our study, maintained oxygen extraction and unaltered transorgan lactate balances speak against the importance of shunting due to 'weak microcirculatory units' [2].

## Conclusion

We conclude that hepatic mitochondrial function in the present model was significantly impaired by endotoxin infusion, without a decrease in microcirculatory blood flow or a deficiency in oxygen extraction. The absence of similar mitochondrial abnormalities in the skeletal muscle and the kidney suggests that the liver has an increased susceptibility to endotoxin-mediated effects.

## Key messages

•Prolonged endotoxemia impairs the liver mitochondrial respiration in the presence of stable regional and microcirculatory blood flow.    

•During endotoxemia, kidney and muscle mitochondrial respiration remain intact.    

•The liver has an increased sensitivity to endotoxin-mediated effects, compared with nonvital organs.   

## Abbreviations

ADP:O = ADP added/oxygen consumed; BSA = bovine serum albumin; L/P = lactate/pyruvate ratio; RCR = respiratory control ratio; TMPD = *N*,*N*,*N*',*N*'-tertamethyl-*p*-phenyldiamine.

## Competing interests

The authors declare that they have no competing interests.

## Authors' contributions

FP participated in the study design and the *in vivo *experiments, carried out the isolation of the mitochondria and wrote the manuscript. JT participated in the study design and the analysis and interpretation of the results, and helped to write the manuscript. CW participated in the *in vivo *experiments, in the preparation of the substrates and in the coordination of the study. HB coordinated the *in vivo *experiments, was responsible for the surgical preparation and participated in the data analysis. AK participated in the *in vivo *experiments. BHL taught the group how to isolate liver mitochondria and helped to write the manuscript. EB participated in the second set of *in vivo *experiments and performed the data analysis. SMJ conceived the study, participated in the study design, the *in vivo *experiments and the analysis and interpretation of the results, and helped to write the manuscript. All authors read and approved the final manuscript.

## Figures and Tables

**Table 1:** Summary of hemodynamic data of the endotoxin-infused and control groups

Parameter	Group	Baseline	1.5 hours	3 hours	6 hours	12 hours	End of experiment
Heart rate (beats/minute)	Endotoxic	115 (90–156)	125.5 (97–139)	132 (103–166)	125 (96–157)	117 (94–171)	109 (98–181)^b^
	Control	101 (82–132)	107 (87–141)	111 (82–127)	103 (78–136)	100 (70–127)	107 (80–117)
Cardiac output (ml/kg/minute)	Endotoxic	92 (61–146)	93 (61–134)	87 (42–143)	102 (66–165)	115 (58–149)^a^	108 (74–152)
	Control	77 (60–143)	86 (55–160)	91 (53–142)	91 (63–136)	107 (70–147)	103 (89–164)

Data presented as the median (range). ^a^Friedman test first 12 hours, *P *< 0.05. ^b^Mann–Whitney *U *test first 12 hours vs control, *P *< 0.05

**Table 2:** Mitochondrial respiration and maximal ATP production in the muscle and the kidney at the end of the experiment

Substrate	Group	Muscle		Kidney	
		
		*n*	Median (range)	*n*	Median (range)
Glutamate					
State 3	Endotoxic	8	174 (93–114)	7	93 (56–129)
	Control	9	223 (67–494)	8	116 (49–165)
State 4	Endotoxic	8	18 (13–42)	7	22 (13–40)
	Control	9	20 (11–49)	8	18 (11–27)
Maximal ATP production	Endotoxic	8	328 (187–940)	7	184 (74–431)
	Control	9	606 (153–1233)	8	221 (133–485)
Respiratory control ratio	Endotoxic	8	8.1 (6.7–11.8)	7	4.0 (2.3–6.3)
	Control	9	8.3 (6.0–14.5)	8	5.4 (3.7–7.1)
Succinate					
State 3	Endotoxic	8	269 (167–467)	7	194 (129–269)
	Control	9	307 (129–579)	8	230 (107–269)
State 4	Endotoxic	8	56 (36–82)	7	67 (33–98)
	Control	9	62 (31–129)	8	60 (36–78)
Maximal ATP production	Endotoxic	8	513 (356–944)	7	460 (169–707)
	Control	9	611 (421–1164)	8	469(193–1292)
Respiratory control ratio	Endotoxic	8	4.9 (2.9–7.0)	7	3.3 (2.0–3.9)
	Control	9	4.1 (3.6–8.8)	8	3.6 (3.0–3.9)
Ascorbate/*N*,*N*,*N*',*N*'-tertamethyl-*p*-phenyldiamine					
State 3	Endotoxic	8	271 (189–401)	7	134 (98–191)
	Control	9	316 (156–412)	8	150 (116–401)
State 4	Endotoxic	8	151 (100–200)	7	91 (49–134)
	Control	9	147 (76–254)	8	99 (62–169)
Respiratory control ratio	Endotoxic	8	1.9 (1.4–2.0)	7	1.6 (1.4–2.7)
	Control	9	1.7 (1.4–2.9)	8	1.8 (1.5–2.8)

**Table 3:** Regional flows, microcirculation, and systemic and regional oxygen extraction and consumption

	Group	Baseline	1.5 hours	3 hours	6 hours	12 hours	End of experiment
Renal blood flow (ml/kg/minute)	Endotoxic	7.7 (2.3–10.5)	7.3 (2.2–11.6)	6.2 (2.4–9.5)	6.1 (2.4–10.0)	5.8 (2.7–10.8)	3.9 (0.9–10.8)^b^
	Control	5.7 (1.9–9.5)	5.6 (2.4–8.2)	5.6 (2.9–7.6)	6.0 (2.9–8.1)	6.3 (3.0–7.9)	5.1 (3.3–8)
Total hepatic blood flow (ml/kg/minute)	Endotoxic	23.2 (13.5–29.3)	22.7 (16.7–27.6)	25.5 (14–36.9)	26.9 (23.3–31.6)	26.7 (21.1–40)^a^	24.5 (19.7–39.6)
	Control	21.5 (17.5–30)	23.7 (17.5–25)	24.8 (18.6–28)	22.5 (21.3–28.1)	26.1 (17.5–29)	25.3 (23.5–42.3)
Liver blood perfusion units (%)	Endotoxic	100	104 (58–229)	111 (48–212)	97 (51–238)	86 (27–212)	70 (22–194)
	Control	100	112 (62–156)	122 (53–221)	94 (43–282)	94 (65–376)	93 (27–251)
Renal blood perfusion units (%)	Endotoxic	100	101 (28–170)	100 (37–141)	89 (31–151)	93 (42–132)	45 (27–102)^b^
	Control	100	71 (35–200)	93 (32–157)	62 (22–171)	90 (20–617)	55 (16–660)
Systemic VO_2 _(ml/kg/minute)	Endotoxic	6.5 (4.7–11.7)	7.1 (3.8–10.7)	7.7 (4.2–11.1)	7 (5.3–10.1)	7.8 (4.9–8.9)	6.9 (4.6–9.3)
	Control	5.2 (4.5–8.4)	6.4 (3.9–7.9)	7.1 (3.2–8.1)	6.1 (3.6–8.9)	7.0 (3.9–8.2)	6.0 (3.8–8.1)
Hepatic VO_2 _(ml/kg/minute)	Endotoxic	0.9 (0.3–1.5)	1.0 (0.5–1.5)	1.1 (0.5–2.6)	1.3 (0.7–2.1)	1.1 (0.6–1.8)	1.1 (0.4–1.8)
	Control	1.0 (0.8–2.0)	1.0 (0.9–1.2)	1.0 (0.7–1.2)	1.1 (0.9–1.5)	1.1 (0.8–1.4)	1.1 (0.7–1.7)
Renal VO_2 _(ml/kg/minute)	Endotoxic	0.2 (0.1–0.3)	0.2 (0.1–0.3)	0.2 (0.1–0.3)	0.2 (0.1–0.3)	0.3 (0.1–0.3)	0.2 (0.04–0.3)
	Control	0.2 (0.1–0.3)	0.2 (0.1–0.2)	0.2 (0.1–0.2)	0.2 (0.2–0.2)	0.2 (0.2–0.3)	0.2 (0.1–0.3)
Systemic oxygen extraction (%)	Endotoxic	57 (43–71)	56 (41–74)	63 (39–73)	62 (47–70)	55 (42–66)	60 (50–88)
	Control	57 (37–65)	56 (45–66)	59 (43–69)	57 (38–70)	54 (39–63)	50 (30–70)
Hepatic oxygen extraction (%)	Endotoxic	46 (35–82)	49 (21–78)	57 (27–84)	60 (33–74)	54 (34–66)	61 (30–74)
	Control	60 (53–73)	62 (39–70)	58 (29–74)	56 (45–78)	61 (34–72)	53 (30–75)
Renal oxygen extraction (%)	Endotoxic	24 (17–37)	21 (18–41)	24 (20–30)	26 (25–53)	29 (21–42)^a^	36 (28–82)^b^
	Control	25 (17–60)	22 (15–55)	24 (17–52)	26 (19–54)	24 (21–58)	30 (24–52)

Data presented as the median (range). ^a^Friedman test first 12 hours, *P *< 0.01. ^b^Wilcoxon test, *P *< 0.05 vs 12 hours.

**Table 4:** Arterial lactate; systemic and regional lactate/pyruvate ratios; regional lactate exchange

	Group	Baseline	1.5 hours	3 hours	6 hours	12 hours	End of experiment
Arterial lactate (mmol/l)	Endotoxic	0.8 (0.4–1.2)	1.0 (0.5–1.3)	0.9 (0.7–1.7)	0.9 (0.7–1.1)	0.8 (0.5–1.0)	0.9 (0.6–4.2)
	Control	0.9 (0.7–1.2)	0.7 (0.5–1.2)	0.7 (0.5–1)	0.7 (0.5–1.0)	0.5 (0.4–0.8)	0.5 (0.3–1.1)
Systemic lactate/pyruvate ratio	Endotoxic	9.4 (6.7–17.0)	9.5 (7.1–18.3)	11.0 (6.7–23.7)	8.9 (7.5–14.1)	9.5 (6.6–10.4)	11.9 (8.5–27.1)^a^
	Control	9.5 (5.7–13.6)	8.1 (5.4–10.0)	8.7 (7.1–10.9)	8.7 (7.2–11.3)	8.5 (5.4–11.8)	7.3 (5.6–11.6)
Hepatic lactate/pyruvate ratio	Endotoxic	9.7 (7.3–27.6)	10.3 (5.7–19.8)	10.7 (6.9–18.6)	9.4 (6.2–18.1)	7.9 (6.2–16.7)	12.1 (7.5–33.0)
	Control	12.4 (3.9–46.9)	9.2 (4.5–26.7)	9.9 (6.7–34.9)	8.2 (5.4–28.3)	7.0 (4.9–30.8)	9.2 (5.8–25.3)
Renal lactate/pyruvate ratio	Endotoxic	8.0 (6.0–10.0)	8.4 (5.5–35.9)	9.8 (6.6–12.5)	8.0 (5.4–15.7)	7.4 (6.3–9.8)	8.1 (7.3–33.6)
	Control	8.7 (6.5–11.0)	7.9 (5.7–13.1)	8.6 (6.1–14.4)	8.7 (5.2–36.1)	7.3 (5.8–37.3)	8.7 (5.3–14.2)
Renal lactate exchange (μmol/minute)	Endotoxic	1.4 (–1.5–2.1)	1.0 (–12.1–1.6)	0.9 (–3.2–2.4)	0.6 (–0.9–1.2)	0.8 (–0.7–2.6)	1.1 (–7.8–2.4)
	Control	0.7 (–3.5–1.6)	0.1 (–4.5–1.5)	0.3 (–1.7–1.3)	0.6 (–11.1–1.3)	0.5 (–9.9–1.5)	0.3 (–1.5–1.4)
Hepatic lactate exchange (μmol/kg/minute)	Endotoxic	9.0 (5.2–20.1)	8.7 (–11.0–22.4)	9.7 (7.4–21.3)	11.6 (9.6–19.0)	12.7 (6.5–18.6)	10.8 (–17.5–15.2)
	Control	10.3 (0.8–18.2)	9.0 (5.9–15.0)	8.6 (3.8–19.0)	7.3 (4.7–16.9)	9.2 (3.5–16.7)	9.4 (5.2–16.7)

Data presented as the median (range).
